# Wanted *Plasmodium falciparum*, dead or alive

**DOI:** 10.15698/mic2015.07.211

**Published:** 2015-07-06

**Authors:** Fatimata Sow, Mary Nyonda, Anne-Lise Bienvenu, Stephane Picot

**Affiliations:** 1 University Claude Bernard Lyon 1, Malaria Research Unit, SMITh, ICBMS, UMR 5246 CNRS-INSA-CPE-UCBL1, 8 avenue Rockefeller, 69373 Lyon cedex 08, France.; 2 Hospices Civils de Lyon, Institut de Parasitologie et de Mycologie Médicale (IP2M), Hôpital de la Croix-Rousse, 103 grande rue de la Croix-Rousse, 69317 Lyon cedex 04, France.

**Keywords:** Plasmodium, malaria, apoptosis, autophagy, cell death

## Abstract

Mechanisms of cell death in unicellular parasites have been subjects of debate for the last decade, with studies demonstrating evidence of apoptosis or non-apoptosis like mechanisms, including necrosis, and autophagy. Recent clarifications on the definition of regulated or accidental cell death by The Nomenclature Committee on Cell Death provides an opportunity to reanalyze some data, re-evaluate conclusions in the light of parasite diversity, and to propose alternative arguments in the context of malaria drug resistance, considering lack of really new drugs in the pipeline. Deciphering the mechanisms of death may help in detection of new drug targets and the design of innovative drugs. However, classifications have been evolving rapidly since initial description of “programmed cell death”, leading to some uncertainty as to whether *Plasmodium* cell death is accidental or regulated.

## INTRODUCTION

Eugene Blank wrote years ago to the Lancet a few words from “The rape of Lucrece” where Shakespeare anticipated the existence of apoptosis 1: *…showing life’s triumph in the map of death, And death’s dim look in life mortality… *But the drama of malaria is far from literature and requires more attention to the mechanisms of parasite death. *Plasmodium falciparum* causes malaria in millions of people each year, killing thousands, and leading to poverty and neurological sequelae for an undetermined number of others 2. Members of the *Plasmodium* genus are more than two hundred, but only five species infect humans: *P. falciparum, P. vivax, P. ovale, P. malariae and P. knowlesi*. Most of the knowledge accumulated over the centuries is on *P. falciparum*. Interest in *P. vivax* is mounting due to several factors, first, it plays a role in severe malaria, second, it develops drug resistance, and third, it has persistent dormant liver forms placing it as a public health problem today and tomorrow 3. Few non-human species have been studied as models on specific aspects of this disease. *P. knowlesi* has been known to humans since 1930, but molecular methods showed more recently that its circulation among humans is more frequent than earlier suspected 4. Extensive knowledge is available about the major steps of the malaria parasite cycle in the human and the mosquito hosts, its biology, and the pathology it induces. On the contrary, very little is known about *Plasmodium* death, natural or induced by a physical, chemical or biological stress. Several mechanisms of cell death are known, including necrosis, apoptosis and autophagy, and these mechanisms have driven an increasing interest over the last two decades.

If a parasite has a “goal”, it is that it be transmitted from host to host. Being responsible for morbidity or mortality is a collateral effect that precludes parasite transmission. High parasite virulence may lead to low transmissibility and sequentially low endemicity, for instance, killing its host before production of sexual forms which can be picked up by mosquitoes for continuity of the cycle, is a cost to parasite fitness. *P. falciparum*, is one of the most harmful of the malaria parasites. One could speculate that it has no inherent limitation to its transmission, leading to devastating disease in humans. On the other hand, one could reason that this unicellular parasite could be acting as a dispersed body within its vector and definitive host, either activating or not mechanisms that could regulate its virulence in terms of infectious burden. The most obvious of these potential mechanisms could be eliminating part of the parasite population in favour of host survival thus sustaining parasite transmission. Regulated cell death may be very well a cornerstone of this strategy.

## *PLASMODIUM* CELL DEATH

Crisis forms of the parasite were described in the early 20^th^ century, but these punctuated and condensed parasites were not extensively studied per se (approximately thirty publications). They were mostly considered as degenerating forms of the parasite induced by the immune response and the “crisis form factor” 5,6. While examining with perplexity *Plasmodium *cultures decreasing in parasitemias under unknown reasons, we wondered whether these crisis forms were still alive. With regards to the first description of apoptosis in a protozoan parasite (*Trypanosoma cruzi*) by Ameisen in 1995 7, we explored the notion that there was more to these crisis forms than parasite corpses. In 1997, we described the apoptosis-like DNA fragmentation of *P. falciparum* parasite in response to chloroquine (CQ) 8. We speculated that resistance to chloroquine was related to inhibition of apoptosis. This first description of apoptosis in *Plasmodium* parasite was not readily accepted by colleagues, and regulated *Plasmodium* death still remains a controversial subject. More than fifteen years later, the debate is still ongoing, and requires to be revisited in light of recent progresses and issues. Among the current issues, recent evidence of artemisinin drug resistance of malaria parasites is a huge matter of concern. Artemisinin and its derivatives may act in part through the induction of parasite cell death via a redox mechanism. A parallel was made decades ago between resistance to regulated cell death (RCD) of cancer cells and choloroquine-resistance. The same paradigm could be used again for artemisinin-resistant parasites, with several issues to be addressed before global spreading of malaria parasite resistance.

## “TO DIE, TO SLEEP — NO MORE…”

The Nomenclature committee on cell death (NCCD) is working to clearly delineate the boundaries between the various pathways leading to death of eukaryotic cells, this is in agreement with continuous progresses made on the mechanisms involved in metazoans 9,10. Their work is useful to better knowledge in eukaryotic parasite life or death decisions. NCCD recently defined cell death as “accidental” or “regulated” 10. Accidental cell death is caused by severe physical, chemical or mechanical insults. It is immediate and without possibility of prevention or regulation by drugs or genetics. It leads to the release of large amounts of damage-associated molecular patterns (DAMP). DAMPs are key danger signals that initiate an immune response and a pro-inflammatory cascade by activating Toll-like receptors (TLR) 11. These TLR activators can also contribute to the pathogenesis of inflammatory diseases and cancer 11. This model of accidental cell death of parasites could be used to define the death of microfilariae when treatments with full doses of Diethyl-carbamazine or ivermectine are used to treat patients presenting a high numbers of parasites.

RCD is based on the activity of complex molecular machinery, giving time to a competitive interplay between mechanisms involved in survival and those involved in death processes. Necrosis is neither the representative form for accidental cell death, nor apoptosis that for programmed cell death. The NCCD stressed the fact that RCD may present both apoptotic and necrotic traits, also that “autophagic cell death” should be used only from a functional perspective, and when RCD can be modulated by drugs or genetic intervention 12. Features of autophagy are frequent during the different RCD processes, but their contribution to cellular demise seems to be limited 13,14. Apoptosis is defined as a caspase dependant variant of RCD, triggered by intrinsic or extrinsic events. Apoptosis relies on the mitochondrial outer membrane permeabilization (MOMP) which requires activity of one of the pro-apoptotic BCL2 family member genes, balanced by the activity of anti-apoptotic members of the same family, under the control of BH3-only proteins. Apoptosis is highly controlled, thus reversible, as demonstrated by the role of metabolic check-points in their attempt to re-establish homeostasis 15. Bcl2-like proteins may operate as central checkpoints of a network of “firebreaks” leading to “the point-of-no-return”. Necroptosis is defined as a capase-independent RCD initiated by death receptors. Parthanatos (caspase-independent cell death involving hyper-activation of poly (ADP-ribose) polymerases and AIF release) and ferroptosis (cell death induced by iron-dependant ROS accumulation through depletion of the antioxidant glutathione in neurons and cancer cells) are other forms of specific mechanisms leading to RCD 10.

It became clearer that cell adaptation to stress is a long-standing mechanism, with the potential of successive initiation of pathways with balanced effects, leading to or not the execution of the death machinery. Interestingly, two exclusive models of cell stress adaptation have been proposed. The conversion model described the succession of RCD-inhibitory signals after the insult, followed by RCD-promoting signals without overlap. The competition model speculates that the pro- and anti-RCD machineries are acting at the same time to favour either cell survival or cell death. This second model seems to be the most reliable for various conditions. These concepts, recently formulated and suspected by many researchers, may explain the abundance of the debate on the “who kills the cell?” scientific question.

Is it expected that new definition will lead to closure on the debate on how *Plasmodium* dies: Is it by necrosis, apoptosis, autophagy or some unknown phenomena? Will the clarification provided recently by the NCCD help to define the protozoan, and more specially the *Plasmodium* cell death? Do all these really matter?

## *PLASMODIUM* CRIME SCENE: NECROSIS, APOPTOSIS, AUTOPHAGY?

In a recent opinion article, Proto and colleagues argued that protozoan cell death (including *Leishmania*, *Trypanosoma*, and *Plasmodium*) should only be considered as incidental cell death or unregulated necrosis 16. Their opinion was mostly based on the fact that definitive evidence of machinery similar to eukaryotic cell is lacking in protozoan parasites. They argued that regulated cell death could only be demonstrated if the death process can be delayed or abolished by targeting key signalling or execution pathways 16. But they also suggested that executioners and/or regulators could be specific in parasitic protozoa. This opinion is driven by extensive evidence from experts and seems to be convincing. Nevertheless, since differences between higher eukaryotic cells and protozoan parasites are obvious, should we look for regulated cell death through the same lens and tools used in parasites as those in higher eukaryotes, including humans? Is it acceptable to consider that, during evolution, a unicellular organism has selected a restricted biological armament essential to transmit advantages, abandoning unnecessary, finely regulated pathways, which are required for a multicellular organism to adapt to a considerable variety of external stresses? Finally, should we affirm that a biological evidence of RCD can exist without an already identified biochemical pathway is nonsense?

## PLASMODIUM APOPTOSIS

The first description of *P. falciparum *apoptosis was made at a time when morphological features were the only markers of apoptosis 8. *P. berghei* features of apoptosis including, condensation of chromatin, DNA fragmentation, and externalisation of phosphatidylserine were demonstrated a few years after that 17. Evidence of *Plasmodium* apoptosis was obtained mostly in two different conditions. First, our primary description of apoptosis features in parasites subjected to chloroquine pressure opened the way to test the potential of other drugs to induce parasite apoptosis. Later on, Matthews and co-workers pointed out that the timing of exposure to drugs such as chloroquine has a major role for the detection of apoptosis markers 18. It was also demonstrated that novel stilbene-chalcone hybrids cause stage-specific apoptosis-like death with MOMP in *P. falciparum* ring stages and trophozoites 19.

Second, the parasite cell death was considered by Hurd and co-workers as a response to within-host environmental factor during mosquito’s stages of the parasite. Several papers have provided evidence for expression of “classical” apoptosis markers under “natural” conditions at a parasite population level inside the mosquito.

The apoptosis machinery began to be depicted with the first description of *P. falciparum* metacaspase 1 (PfMCA1) 20. The caspase-like cell death was specific to a CQ sensitive clone. z-VAD-fmk can restore *P. falciparum* proliferation under CQ pressure and reduce the CQ sensitivity of the parasite. Fascinatingly the CQ resistant clone was insensitive to z-VAD-fmk indicating the CQ resistance level could be limited by other mechanisms. More recently, the metacaspase 1 gene was characterized in *P. vivax* isolates 21.

The expression of PfMCA1 C14 peptidase domain in *yca1* deficient *Saccharomyces cerevisiae* led to a growth retardation and a drastic yeast cell death 22. Of significance, this phenotype could be blocked by the addition of the pan caspase inhibitor z-VAD-fmk while PfMCA1 did not exhibit a caspase-like but an arginine protease activity, as reported for other protozoan MCAs 22. PfMCA1 could play an initiator role leading to the activation of an aspartate protease effector. This hypothesis is in agreement with the description of PfMCA1 auto-processing leading to prodomain removal as it is typical of initiator caspases. PfMCA1 function seems to be finely regulated by two putative binding domains described: a C2 calcium-dependant membrane targeting domain and a CARD domain (Caspase Recruitment Domain) 20,22,23. Authors suggested that the clan CA mediators are likely to be the proteases accounting for the cysteine protease activity 24.

Proteins with caspase-like activity were identified in the cytoplasm of the ookinete, and more than 50% of the mosquito midgut stages of the parasite die naturally by apoptosis before gut invasion. This phenomenon was prevented by a caspase inhibitor 17.

Following observations of pro- or contra- apoptosis evidences in *Plasmodium* parasites confirmed that the diversity is a cornerstone of cell death mechanism definition, and that different factors should be taken into consideration during the process of cell death characterization.

## ALL BUT APOPTOSIS!

Using F32 *P. falciparum* clone (CQ sensitive), Nyakeriga *et al. *obtained a disparity of features associated with death induced *in vitro* by treatment with chloroquine, atovaquone, etoposide and L-penicillamine, without a complete panel of apoptosis markers for all drugs 25. They failed to detect DNA degradation and concluded that *Plasmodium* cell death “is not associated with typical feature of apoptosis after treatment, which are characteristic of vertebrate cells”. Using CSC-A (Honduras) *P. falciparum* clone, Porter *et al.* failed to detect typical physiological hallmarks of what they called “classical” apoptosis 26. They concluded that apoptosis could be eliminated as a mechanism of *Plasmodium* death, and they consider that data presented in the first demonstration of *P. falciparum* apoptosis 8 were due to white blood cell contamination of the medium.

While nothing could be excluded definitively 15 years after the experiments were conducted, fresh parasite cultures were newly tested with blood controlled for the absence of leucocytes. Same blood was used to simultaneously cultivate two different clones, and apoptosis hallmarks were only observed with the CQ sensitive clone. Examining blood smears twice a day for weeks would have revealed the suspected contamination by leucocytes. Returning to the raw data, parasitemias obtained during this period were as high as expected, leading to the hypothesis of the absence of leucocytes. Porter and colleagues then postulated that parasites were dying with signs of secondary necrosis, without a clear definition of these mechanism, and also stated that some form of programmed cell death could not be ruled out.

Using PSS1 (Brazil) *P. falciparum* clone (CQ resistant), Totino *et al.* failed to observe apoptosis after treatment with chloroquine and staurosporine, in agreement with our demonstration that drug resistance to CQ may be linked to defect in apoptosis 27. They observed a small percentage of TUNEL-positive parasites that they attributed to a small proportion of CQ sensitive parasite in the PSS1 resistance clone, but long-term cultivation could have selected a monoclonal culture. Finally they concluded that parasites were dying through an autophagic mechanism, on the basis of ultrastructural analysis that they considered a gold standard for identification of cell death procedure. Using *P. berghei* ANKA transgenic parasites (mCherry, GFP), Eickel *et al.* showed features of autophagy in liver-stage parasites 28.

## PRO-APOPTOSIS CORNER

Using 3D7 and 7G8 CQ sensitive and resistant *P. falciparum* clones, Picot *et al. *highlighted the first evidence of DNA fragmentation (DNA ladders and TUNEL), MOMP, PS exposure in *P. falciparum* erythrocytic stages 8,20. The same team also described the *P. falciparum* metacaspase and its role in cell death 22. Other studies using *P. berghei* ookinetes by Arambage *et al.* showed evidence of naturally occurring apoptosis in the mid gut of mosquitoes 29. Muregi *et al.* also showed evidence of apoptosis by electron transmission microscopy and internucleosomal DNA fragmentation in parasites resistant to a thymidylate synthase inhibitor (5-fluoroorotate) 30. In addition to these, using a 3D7 CQ sensitive *P. falciparum* clone, Rathore *et al.* elucidated features of apoptosis-like cell death (MOMP, caspase-like activity, dose dependant DNA fragmentation) by interfering with the ClpQY machinery 31. Another study using Dd2 CQ sensitive *P. falciparum* clone by Mutai and Waitumbi showed evidence of DNA fragmentation, MOMP, expression and translation of metacaspase gene under the effect of increasing parasitemia, suggesting existence of a quorum-sensing like mechanism 32. Finally Cheema *et al.* used NF54 CQ sensitive *P. falciparum* clone to show evidence of MOMP, caspase activation and DNA fragmentation induced by Glaridin, a polyphenolic flavonoid 33.

## *PLASMODIUM* DEATH MODE: A QUESTION OF TIME?

Recent work has demonstrated that among the erythrocytic stages of *P. falciparum*, response to heat will lead to apoptosis-like phenotype in the ring-stages, and an autophagy-like phenotype in late-stages of the parasite cycle. This provides more evidence that *Plasmodium* cell death is a fluctuant adaptation to stress, and that it will be difficult to clearly meet the definition criteria of regulated or accidental cell death 34. The variability of the adaptive phenotypes of cell death in response to subtle changes of within-host environment was pointed out a few years ago 35. In the absence of more detailed studies using a broad range of *Plasmodium* clones of different origins and without the aim of demonstrating the relevance of one cell death mechanism compared to others, there are few chances to obtain a balanced evidence of regulated cell death of *Plasmodium* parasite. One of the variables to be tested is the kinetics of *Plasmodium* cell death. It has become clear that the delay between the death message and cell demise is crucial. The complexity of pathways potentially involved could lead to changes in the cellular and biochemical manifestations of parasite death. Depending on the time plan of the tests performed to decipher parasite death mechanisms, markers could be different.

William Shakespeare also wrote “…for there is nothing either good or bad, but thinking makes it so…” and was cited earlier by Lipton and Nakanishi 36. The fantastic debates about mechanisms of *Plasmodium* death is of utmost importance for the development of future antimalarial drugs. Since scientific terminology is evolving rapidly, it could be counterproductive to reduce the mechanisms of death in *Plasmodium* parasite to a strict definition that will be outdated with increase in knowledge. The fight against malaria disease and its unacceptable toll of children and adults deaths requires a rapid development of innovative treatment to address the issues of increasing drug resistance. The Achilles’ heel of the parasite could be its sensitivity to regulated cell death, providing new drug targets that we expected from the first observation of *Plasmodium* DNA fragmentation. There is probably a gap to fulfil between academic research needed to decipher the mechanisms of cell death in *Plasmodium* parasite, and clinical research needed to improve the outcome of malaria patients. Decades ago, Chloroquine and Quinine have been successfully used to treat patient without definitive knowledge on their mechanisms of action. It is tempting to speculate that there is enough evidence of regulated cell death for a translational research with the aim of designing new drugs to specifically kill parasites using the apoptosis-autophagy machinery.
